# Method for the Destruction of Endotoxin in Synthetic Spider Silk Proteins

**DOI:** 10.1038/s41598-018-29719-6

**Published:** 2018-08-15

**Authors:** Richard E. Decker, Thomas I. Harris, Dylan R. Memmott, Christopher J. Peterson, Randolph V. Lewis, Justin A. Jones

**Affiliations:** 1 Department of Biological Engineering, Utah State University, Logan, Utah 84322 United States; 2 Department of Biology, Utah State University, Logan, Utah 84322 United States

## Abstract

Although synthetic spider silk has impressive potential as a biomaterial, endotoxin contamination of the spider silk proteins is a concern, regardless of the production method. The purpose of this research was to establish a standardized method to either remove or destroy the endotoxins present in synthetic spider silk proteins, such that the endotoxin level was consistently equal to or less than 0.25 EU/mL, the FDA limit for similar implant materials. Although dry heat is generally the preferred method for endotoxin destruction, heating the silk proteins to the necessary temperatures led to compromised mechanical properties in the resultant materials. In light of this, other endotoxin destruction methods were investigated, including caustic rinses and autoclaving. It was found that autoclaving synthetic spider silk protein dopes three times in a row consistently decreased the endotoxin level 10–20 fold, achieving levels at or below the desired level of 0.25 EU/mL. Products made from triple autoclaved silk dopes maintained mechanical properties comparable to products from untreated dopes while still maintaining low endotoxin levels. Triple autoclaving is an effective and scalable method for preparing synthetic spider silk proteins with endotoxin levels sufficiently low for use as biomaterials without compromising the mechanical properties of the materials.

## Introduction

The biocompatibility and mechanical properties of spider dragline silk set it apart from most synthetic and natural materials as an ideal biomaterial. Dragline silk is made up of two proteins, MaSp1 and MaSp2^1,2^, whose structures make the resultant fibers strong, extensible, and flexible^3^. With synthetic spider silk proteins, these properties can be harnessed into a variety of materials in addition to fibers, such as films, coatings, gels, and adhesives^4,5^. Synthetic spider silk materials (including fibers) can also be tailored to have increased strength and/or flexibility through mixing different ratios of the dragline proteins. Due to its versatility, synthetic spider silk has great potential for a variety of biomedical applications.

Although native spider silk is generally accepted as biocompatible^6,7^, it is difficult to obtain in large quantities because spiders can only produce a limited amount of silk in a day and cannot be farmed efficiently due to their territorial and cannibalistic nature. In response to these issues, synthetic spider silk proteins have been produced in a variety of hosts^8,9^, including yeast^10^, bacteria^11,12^, silkworms^13,14^, tobacco plants^15^, and goats^16^. While each of these expression systems has advantages and disadvantages for producing spider silk proteins, all are subject to contamination with environmental pyrogens, especially endotoxins, during production and processing.

Pyrogens are substances that produce a fever that can quickly become dangerously high. Endotoxins, one of the most prevalent pyrogens, are surface lipopolysaccharides (LPS) released from gram-negative bacteria that are heat stable to high temperatures^17^. As with all pyrogens, if endotoxins are present at high enough levels, they will induce a fever^18^. Because of this, endotoxin level testing and subsequent removal or destruction is required for any implantable biomaterial^19,20^. Although endotoxins are produced by gram-negative bacteria, they can be found in most environments, particularly on a goat farm and in a laboratory environment^21^.

The most common method for pyrogen destruction (depyrogenation) of materials such as packaging and medical devices is dry heat. Materials to be depyrogenated are treated at 250 °C for ≥30 min^17,22^. The temperature can be adjusted, but it cannot be lower than 180 °C, and at that temperature the samples must be treated for ≥3 hours^22^. Unfortunately, most proteins and other polymers cannot withstand these high temperatures. Although triple autoclaving has been proposed as a potential method for removing endotoxin, the effectiveness of this and other alternative methods of depyrogenation, including caustic rinses, is often debated^17^.

Common methods for removing endotoxin from solutions, such as size-exclusion or ion-exchange chromatography, are effective for medical injectables and microbial-produced bioproducts such as small (<100 kDa), soluble proteins. Although spider silk can be solubilized under specific conditions, it is likely to solidify as the conditions change during the endotoxin removal processes. Ion-exchange chromatography often leads to an increase in salt concentrations, which are detrimental to the formation of spider silk materials^23^, and would require additional washing steps for the silk proteins. Arguably the most significant problem with these methods of endotoxin removal is that they are most effective on smaller soluble proteins. The mechanical properties of spider silk materials are highly dependent on the size of the protein – larger (>100 kDa) is better^15,24,25^. Due to these issues, common methods for removing endotoxins cannot be used on silk proteins.

In light of this, a method to destroy or remove endotoxins from synthetic spider silk (or the materials produced from them) is needed to produce biocompatible and implantable materials. To our knowledge, there is no such method that has been reported in literature. To remedy this, we sought to determine the best method to either remove or destroy endotoxins present in synthetic spider silk proteins and synthetic spider silk protein-based materials while maintaining the valuable mechanical properties of the spider silk.

## Results and Discussion

As can be seen in Table 1, it is possible to decrease the endotoxin levels of synthetic spider silk protein by autoclaving three times. Dry heating also decreased the endotoxin level (data not shown), but even at the lowest acceptable treatment temperature of 180 °C the recombinant spider silk’s mechanical properties were compromised. Protein treated with dry heat made very poor films that could not be tested because they broke when handled. Similarly, dry heated fibers also became very brittle. This decrease in mechanical properties is likely due to the extreme dehydration and resultant degradation of the spider silk that occurs at high temperatures and/or pressures^23^. The dry heated protein also had significant discoloration (brownish-yellow or black), indicating that it had been charred. Due to the detrimental effects of dry heating on the mechanical properties of the samples, the dry heat treatment method was discarded. In contrast, the autoclaved protein samples maintained their color and the resultant films had mechanical properties similar to films made from untreated proteins (Table 2). It is also of interest that films maintained decreased endotoxin levels when they were made on endotoxin-free polydimethylsiloxane (PDMS) molds, but not when made on untreated PDMS molds (Supplementary Table S1, Sample 15). This further confirms that autoclaving destroys the endotoxin in the silk proteins.

**Table 1:** Combined average endotoxin levels of all goat-derived spider silk proteins and films before and after treatments.

Sample Type	Treatment	Results (EU/mL)	n
Protein Powder	None	5.92 ± 0.07	2
Film	NaOH rinse + H_2_O rinse	2.08 ± 0.11	2
Film	NaOH rinse + H_2_O rinse	4.05 ± 0.42	2
Protein Powder	Doping	3.20 ± 1.71	16
Protein Powder	Doping + Autoclaving × 3	**0.17 ± 0.09**	14
Films	Doping + Film	0.81 ± 0.36	4
Films	Doping + Autoclaving 3X + Film	**0.18 ± 0.07**	11

Individual sample group averages are shown in Supplementary Table S1. The R^2^ of the endotoxin analysis kit standard curve was ≥0.989 for all experiments. Silk samples below 0.25 EU/mL are in bold. Standard deviations were calculated using STDEV.P in Microsoft Excel.

**Table 2:** Data from tensile tests on films made from triple autoclaved and untreated synthetic spider silk dopes.

Sample	Group	Average Maximum Stress (MPa)	Average Maximum Strain (%)	Average Maximum Toughness (MJ/m^3^)	n
Unstretched MaSp1	Untreated	39.1 ± 21.2	1.8 ± 0.2	0.33 ± 0.2	6
	A	41.7 ± 3.8	1.7 ± 0.3	0.32 ± 0.08	4
	B	21.5 ± 7.1	1.6 ± 0.1	0.16 ± 0.04	2
	C	52.3 ± 6.1	1.9 ± 0.2	0.45 ± 0.08	4
Unstretched Resolubilized MaSp1	Not Centrifuged	73.9 ± 15.2	2.3 ± 0.3	0.82 ± 0.31	5
	Centrifuged	45.9 ± 10.2	2 ± 0.3	0.47 ± 0.14	3
Unstretched MaSp2	Untreated	189.1 ± 25	3.2 ± 0.4	3.06 ± 0.54	4
	Autoclaved	134.1 ± 29	2.5 ± 0.6	1.69 ± 0.82	4
Stretched MaSp2	Untreated	104.7 ± 32.3	57 ± 5	50.65 ± 10.55	4
	Autoclaved	79.2 ± 17.8	23 ± 11	15.24 ± 6.63	3

Groups A, B, and C were made from different dopes of the same protein; all received the same 3x autoclave treatment. All MaSp1 sample groups were created from dopes made from the same goat-derived protein stock, but at different times. MaSp2 samples were made from dopes of bacterially-derived protein. Standard deviations were calculated using STDEV.P in Microsoft Excel.

Only protein powder, dopes (protein solubilized in water using microwave irradiation to generate high heat and pressure; for details see Methods), and fibers were treated via autoclaving because films deformed (melted) when autoclaved and could not be tested. All film samples presented here were made from autoclaved dopes.

Although triple autoclaving did not cause fibers to become as brittle as dry heated fibers, which broke when handled, the autoclaved fibers still had a significant drop in mechanical properties. It was possible to handle most of the autoclaved fibers to prepare them for mechanical testing, but the samples broke before any meaningful data could be gathered during testing. Some samples were autoclaved immersed in water to test whether dehydration was the sole cause of the increased fragility, but the result was the same, indicating that the issue could be due to the combination of high temperature and high pressure over time.

Because early tests indicated that it would be necessary to eliminate endotoxins in synthetic spider silk fibers (data not shown), dry heat and autoclaving treatments were investigated. While it was found that autoclaving did decrease the endotoxin level in most fiber samples, the results were not as consistent as those of dopes and film samples.

Unlike fibers, the mechanical properties of silk films were consistent between films made from untreated dopes and autoclaved dopes (Table 2). Generally, stretching films increases the mechanical properties of spider silk films^5^. In this case, the average ultimate tensile stress of the films was decreased due to stretching, but the strain was greatly increased. It is possible that this was due to the degree of stretching used or due to the stretched samples being composed completely of MaSp2. Native spider dragline silk and many synthetic spider silk films contain at least some MaSp1, which contains additional strength producing crystalline motifs^3^. Although autoclaving has been shown to affect the structure of silkworm silk^26,27^, Hedhammer *et al*. showed that one round of autoclaving spider silk did not affect the structure of the silk^28^. It is also very likely that any structural changes that may have occurred due to autoclaving are “reset” when the protein is redoped after autoclaving. This lack of structural difference not only explains the similar mechanical properties of the treated and untreated films, but is also beneficial when comparing treated and untreated proteins, as they are more analogous to each other.

The resolubilization “treatment” referenced in Table 2 consisted of triple autoclaving protein powder in water, then, following autoclaving, centrifuging the protein-water mixture, removing the water, and freezing the pellet, or simply freezing the autoclaved protein-water mixture. Both samples were then lyophilized and redoped. This was done to determine whether it would be possible to create stocks of endotoxin-free protein and whether it was better to remove the water previous to freezing and subsequent lyophilization or not. As can be seen in Table 2, the films made from dopes that received the resolubilization treatment had mechanical properties similar to films made from freshly treated dopes. This indicates that it is possible to create stocks of endotoxin-free synthetic spider silk that can be used at future times to make endotoxin-free materials. Unfortunately, the practicality of maintaining an endotoxin-free “common stock” in a laboratory setting is that the stock will not stay endotoxin-free for very long. However, in certain conditions, such as a clean room, good laboratory practice, or good manufacturing practice, an endotoxin-free stock may be very practical and beneficial. Based on the “Not Centrifuged” and “Centrifuged” samples, autoclaving, freezing, and lyophilizing without removing the water from the dope may give better mechanical properties than centrifuging the dope and removing the water before freezing and lyophilizing.

Most of the samples tested in this experiment were from goat-derived spider silk protein. Ideally, synthetic spider silk will be produced predominately in *E*. *coli*. Hedhammar et al. were able to create fibers from small recombinant spider silk proteins produced in *E*. *coli* with low pyrogenicity by treating the bacteria with a combination of Tris, Ca^2+^, and EDTA before cell lysis and subsequent silk extraction^28^. While this method of endotoxin removal is beneficial for a bacterial production system, it is still difficult to produce native size spider silk proteins in bacteria. Because of this, there is great benefit in developing a method of endotoxin removal/destruction that can be applied to synthetic silk proteins regardless of the production system. In this study, a batch of bacterially-derived spider silk protein was treated by triple autoclaving. Results indicate that the treatment method also effectively removes endotoxin in the bacterially-derived silk (Table 3). It is notable that autoclaving silk proteins is more easily scalable and likely less expensive than the endotoxin removal method developed by Hedhammar et al., as autoclaving does not require any reagents or extra purification steps.

**Table 3:** Endotoxin levels of bacterially-derived protein and subsequent films that were either treated with autoclaving or untreated.

Sample Type	Sample	Treatment	Results (EU/mL)
Empty vial	B1	None	0.05 ± 0.00
Empty vial	B2	Autoclave x3	0.06 ± 0.00
Powder	B3	None	2.28 ± 0.02
Film	B4	Doping + Film	2.20 ± 0.01
Film	B5	Doping + Film	2.29 ± 0.01
Powder	B6	None	2.25 ± 0.01
Film	B7	Doping + Autoclave × 3 + Film	**0.185 ± 0**
Film	B8	Doping + Autoclave × 3 + Film	**0.178 ± 0.01**

Empty vials were vortexed with endotoxin-free water in them and the water was tested. The R^2^ of the standard curve was 0.9913 for all tests. All samples had n = 2 except B7, which had n = 1 due to contaminants in the testing well that interfered with the absorbance reading. Silk samples below 0.25 EU/mL are in bold. Standard deviations were calculated using STDEV.P in Microsoft Excel.

## Conclusion

Treating synthetic spider silk protein dopes with three consecutive autoclave cycles is an effective method for reducing endotoxin levels. Autoclaving at the dope stage greatly reduces endotoxin levels without destroying the protein-based materials or compromising their mechanical properties, thus yielding the best combination of endotoxin level reduction and mechanical properties in the final products. This combination will allow synthetic spider silk research to progress to meaningful biocompatibility testing and, eventually, clinical studies without the concern of endotoxin as a confounding factor.

## Methods

### Preparation of Silk Samples

Most samples were made from goat-derived MaSp1 recombinant spider silk protein. Silk proteins were extracted from goat milk and formed into either films or fibers using the aqueous method described previously^5,29^. Briefly, silk proteins are removed from defatted goat milk via tangential flow filtration, precipitation, washing, and subsequent lyophilization. Silk protein solutions, or dopes, are then made by mixing the dry protein with water and microwaving the mixture in a tightly sealed vial in 5–10 s bursts to achieve a minimum temperature of 120 °C under high pressure to solvate. All dopes used in this study were 5% (w/v) protein (150 mg protein in 3 mL water). Dopes were then either poured onto PDMS molds to form films or spun into fibers using a custom “wet spinning” spin line^5,29^.

Final samples were either a full film (30 mm × 6 mm × 50 µm), a six fiber bundle ($\sim$2 cm length, $\sim$3 µm diameter), or just protein powder. Powder samples were prepared by vigorously vortexing 150 mg of protein powder in 3 mL of endotoxin-free water for >5 min. The mixture was then centrifuged at 4,185 × g for 10 min, after which 1 mL of the supernatant was removed and stored for each sample. Samples were also taken after doping following the same procedure used for the powders. For all sample sets, control samples were taken prior to any treatments (including doping) and negative controls (endotoxin-free water) were included.

Some samples were made from bacterially-derived MaSp2 recombinant spider silk protein. Silk proteins were produced in *Escherichia coli* and subsequently extracted as previously described^30^. The proteins were then doped and samples were prepared as described above.

As a note, the doping process is sufficient to sterilize the proteins for cell culture work. Because of this, the doping process was tested for its effectiveness in destroying endotoxin. It was shown that any reduction in endotoxin levels caused by microwaving was insignificant (data not included), so other treatment methods were still required.

### Endotoxin Removal/Destruction

Multiple methods of destroying endotoxins from samples were tried in this study: dry heat, caustic washes, water washes, and autoclaving. All equipment used for handling, storing, and preparing samples was depyrogenated via dry heat at 250 °C for at least 30 min.

The dry heat treatment involved heating samples to 250 °C for at least 30 min or 180 °C for at least 3 hours. During heating, all samples were placed in a loosely covered glass container. After heating, samples were covered and stored in a sterile PCR hood until ready for use. Treated samples were only handled with endotoxin-free equipment, as confirmed through the use of the preferred endotoxin detection kit used in these experiments (data not included).

Caustic washes were performed with 1 M sodium hydroxide (NaOH). Treatments were performed on dry spider silk samples by soaking in 40 mL of NaOH for 3 min. The NaOH was then removed via centrifugation at 4,185 x g and the samples were rinsed with endotoxin-free water three to five times, which was also removed via centrifugation. Samples were then stored in a small amount of endotoxin-free water until testing.

Autoclaving was performed on silk samples using a standard 20 min liquid cycle (121 °C, 15 psig, 1 min purge). The cycle was repeated three times. The door was opened for 1 min between each cycle to allow the autoclave pressure sensor to return to atmospheric pressure before proceeding.

Protein powder was mixed with water and then autoclaved in loosely capped bottles. After autoclaving, water was removed from powder samples via centrifugation at 4,185 x g and subsequent pipetting. Dopes were made as described above then transferred to a clean vial that was loosely capped for autoclaving. After autoclaving, the protein powder and dope samples were doped or redoped, respectively, before testing or casting films.

### Film Stretching

Some films were stretched before mechanical testing (Table 2). Stretching is a common treatment performed on synthetic spider silk fibers and films to improve their mechanical properties^5,29^. In this study, films were stretched in an 80:20 isopropanol:water bath to three times their original length using a stretching apparatus developed by Tucker *et al*.^5^. Films were allowed to dry before being removed from the stretching apparatus and tested. The stretching was not performed under endotoxin-free conditions.

### Resolubilization

To test whether it would be possible to make large stocks of endotoxin-free protein powder for future use without the silk losing its mechanical properties, two processes were tested on dopes that had been autoclaved three times: (1) the autoclaved dope was centrifuged at 4,185 × g, the supernatant was removed, and the protein pellet was frozen; (2) the entire autoclaved dope was frozen. The frozen samples were then lyophilized, after which the protein was resolubilized in water. Films were then made using the standard procedure outlined above.

### Endotoxin Level Analyses

The preferred kit to determine the endotoxin levels for these experiments was the Pierce Limulus Amebocyte Lysate (LAL) Chromogenic Endotoxin Quantitation Kit (Thermo Scientific Cat# 88282), which uses UV absorbance at 405–410 nm to determine endotoxin concentration. This kit has a working detection range of 0.1–1.0 EU/mL. Endotoxin levels above 1.0 EU/mL were extrapolated using an experimentally determined standard curve equation.

Because testing a solid piece of silk material interfered with the UV absorbance and confounded the endotoxin level readings, 1 mL of endotoxin-free water was added to samples after endotoxin destruction treatments. Sample/water mixtures were vigorously vortexed for >5 min to break apart the silk material and remove endotoxin from the silk and the container into the water; 50 µL of the water was then used for endotoxin testing.

### Data availability

The authors declare that the materials, data, and associated protocols used to produce the results presented in this manuscript will be made available promptly to the Editorial Board Members, Referees, and readers upon request.

## Electronic supplementary material

Supplementary Information
