# Supplementary material for: Method for the Destruction of Endotoxin in Synthetic Spider Silk Proteins
Source: Sci Rep. 2018 Aug 15;8:12166. doi: 10.1038/s41598-018-29719-6 (PMC6093939; doi:10.1038/s41598-018-29719-6)
Supplement: Supplementary file 1 — Supplementary Information [file 41598_2018_29719_MOESM1_ESM.pdf]

Method for the Destruction of Endotoxin in Synthetic Spider Silk Proteins

Richard E. Decker[†], *Thomas I. Harris*[†], *Dylan R. Memmott*[‡], *Christopher Peterson*[‡], *Randolph V. Lewis*[‡], *and Justin A. Jones*[*,‡]

Departments of †Biological Engineering and ‡Biology, Utah State University, Logan, Utah 84322, United States

Supplementary Table S1. Endotoxin levels of goat-derived synthetic spider silk protein treated with various endotoxin destruction techniques. Although all protein came from the same stock, horizontal lines are used to separate different sample batches that were treated and tested at different times. The R^2 of the standard curve was ≥ 0.989 for all experiments. Silk samples below 0.25 EU/mL are in bold. Sample 9 had n=2 due to a negative absorbance on one of the sample replicates that was excluded from the average. Sample 15 was made on untreated PDMS. Standard deviations were calculated using STDEV.P in Microsoft Excel.

Sample Type	Sample	Treatment	Endotoxin Level (EU/mL)	n
Powder	1	None	5.02 ± 0.07	2
Film	2	NaOH rinse + H_20 rinse	2.08 ± 0.11	2
Film	3	NaOH rinse + H_20 rinse	4.05 ± 0.42	2
Powder	4	Doping	5.20 ± 0.24	3
Powder	5	Doping + Autoclave x3	0.307 ± 0.10	3
Film	6	Doping + Autoclave x3 + Film	**0.249 ± 0.08**	3
Powder	7	Doping	1.73 ± 0.10	3
Powder	8	Doping + Autoclave x3	**0.063 ± 0.01**	3
Film	9	Doping + Autoclave x3 + Film	**0.103 ± 0.01**	2
Powder	10	Doping	1.99 ± 0.05	3
Powder	11	Doping + Autoclave x3	**0.115 ± 0.00**	3

Film	12	Doping + Autoclave x3 + Film	**0.123 ± 0.06**	3
Powder	13	Doping	1.79 ± 0.13	3
Powder	14	Doping + Autoclave x3	**0.098 ± 0.01**	3
Film	15	Doping + Autoclave x3 + Film	1.62 ± 0.03	3
Powder	16	Doping	2.23 ± 0.02	2
Film	17	Doping + Film	1.17 ± 0.02	2
Film	18	Doping + Film	0.446 ± 0.02	2
Powder	19	Doping	2.28 ± 0.01	2
Powder	20	Doping + Autoclave x3	**0.136 ± 0.01**	2
Film	21	Doping + Autoclave x3 + Film	**0.202 ± 0.02**	2
Film	22	Doping + Autoclave x3 + Film	**0.214 ± 0.00**	2
